# The FBXW7-binding sites on FAM83D are potential targets for cancer therapy

**DOI:** 10.1186/s13058-024-01795-9

**Published:** 2024-03-07

**Authors:** Xiaoyu Jiang, Yuli Wang, Lulu Guo, Yige Wang, Tianshu Miao, Lijuan Ma, Qin Wei, Xiaoyan Lin, Jian-Hua Mao, Pengju Zhang

**Affiliations:** 1 Key Laboratory Experimental Teratology of the Ministry of Education, Department of Biochemistry and Molecular Biology, School of Basic Medical Sciences, Cheeloo College of Medicine, Shandong University, Jinan, Shandong 250012 China; 2 Department of Clinical Laboratory, The Second Hospital of Shandong University, No. 247 Beiyuan Street, Jinan, Shandong 250033 China; 3 Department of Clinical Pharmacy, College of Pharmacy, Xinxiang Medical University, Xinxiang, Henan 453000 China; 4 Department of Pathology, Shandong Provincial Hospital Affiliated to Shandong University, Jinan, China; 5 Biological Systems and Engineering Division, Lawrence Berkeley National Laboratory, Berkeley, CA USA

**Keywords:** FBXW7, FAM83D, Ubiquitination and degradation, Breast cancer, Metastasis, Chemotherapy

## Abstract

Increasing evidence shows the oncogenic function of FAM83D in human cancer, but how FAM83D exerts its oncogenic function remains largely unclear. Here, we investigated the importance of FAM83D/FBXW7 interaction in breast cancer (BC). We systematically mapped the FBXW7-binding sites on FAM83D through a comprehensive mutational analysis together with co-immunoprecipitation assay. Mutations at the FBXW7-binding sites on FAM83D led to that FAM83D lost its capability to promote the ubiquitination and proteasomal degradation of FBXW7; cell proliferation, migration, and invasion in vitro; and tumor growth and metastasis in vivo, indicating that the FBXW7-binding sites on FAM83D are essential for its oncogenic functions. A meta-evaluation of FAM83D revealed that the prognostic impact of FAM83D was independent on molecular subtypes. The higher expression of FAM83D has poorer prognosis. Moreover, high expression of FAM83D confers resistance to chemotherapy in BCs, which is experimentally validated in vitro. We conclude that identification of FBXW7-binding sites on FAM83D not only reveals the importance for FAM83D oncogenic function, but also provides valuable insights for drug target.

**Supplementary Information:**

The online version contains supplementary material available at 10.1186/s13058-024-01795-9.

## Introduction

Breast cancer (BC) is the most diagnosed cancer among women and the second leading cause of cancer-related death in women [1, 2]. Like other malignant tumors, the initiation and progression of BC are affected by a variety of factors involving multiple oncogenes and tumor suppressor genes. For example, BRCA1 gene mutation leads to accumulation of DNA damage and genome instability, which promotes malignant transformation of breast cells [3]. PI3KCA has a high frequency mutation in BC and is associated with chemotherapy resistance and poor prognosis of BC patients [4–6]. HER2 overexpression activates PI3K/Akt/mTOR signaling pathway and promotes the development of BC [7]. With the growing knowledge of the pathogenesis of BC, targeted therapy such as the application of Olaparib and Aapelicib respectively targeting BRCA and PI3KCA gene mutation, has sprung up in recent years in addition to the systemic treatments including surgery, radiotherapy, chemotherapy, and endocrine therapy, which have significantly improved patient outcomes [8, 9]. However, due to the highly heterogeneous nature of BC, the treatments provide certain benefit in some BC patients and the overall survival rate is still gloomy. Therefore, unraveling novel targets in BC will offer new opportunities in improving treatments for the BC patients.

FAM83D (family with sequence similarity 83, member D, also known as CHICA) is initially identified as a mitosis-associated protein which plays a key role in the processes of cell mitosis, especially in the separation of sister chromatids and the aggregation of chromosome equatorial plates [10]. Recent studies have shown that FAM83D serves as an important oncoprotein tightly linked to carcinogenesis [11–15]. For example, FAM83D is frequently up-regulated in multiple types of cancer such as lung cancer [16], ovarian cancer [13], gastric cancer [17, 18], colorectal cancer [19] and pancreatic adenocarcinoma [11]. Moreover, the elevated expression of FAM83D is positively related to the poor prognosis of most cancers [16, 18, 20, 21]. Our previous study found that FAM83D expression was significantly increased in human BC cells. Knockdown of FAM83D dramatically inhibited the proliferation, invasion, migration and epithelial-mesenchymal transition (EMT) of BC cells, indicating its pro-oncogenic potential in BC [22]. Notably, our study also revealed that FAM83D could bind to FBXW7 physically and downregulated the expression of FBXW7. FBXW7 belongs to the F-box protein (FBP) family and acts as a substrate recognition component of SCF (SKP1/CUL1/F-box) E3 ubiquitin ligase [23]. It is widely known that FBXW7 is a critical tumor suppressor which is commonly inactivated in human lung, gastric, breast and several other cancers through genetic and epigenetic mechanisms, along with post transcriptional modifications [24–27]. Although the data in our previous work implied that FAM83D may exert its oncogenic roles by inhibiting FBXW7, the involvement of FBXW7 in the malignant phenotype of BC cells driven by FAM83D and the detailed mechanism underlying FBXW7 down-regulation induced by FAM83D are still elusive.

In the present study, we further explored the contribution of FBXW7 to the pro-oncogenic activity of FAM83D and deciphered the molecular basis of FAM83D-triggered downregulation of FBXW7. Our findings will be helpful to better understand the role and mechanism of FAM83D in the development and progression of BC as well to guide the personalized treatments of BC patients.

## Results

### The residues H343/L344 of FAM83D contribute to FBXW7 binding

**Fig. 1:**
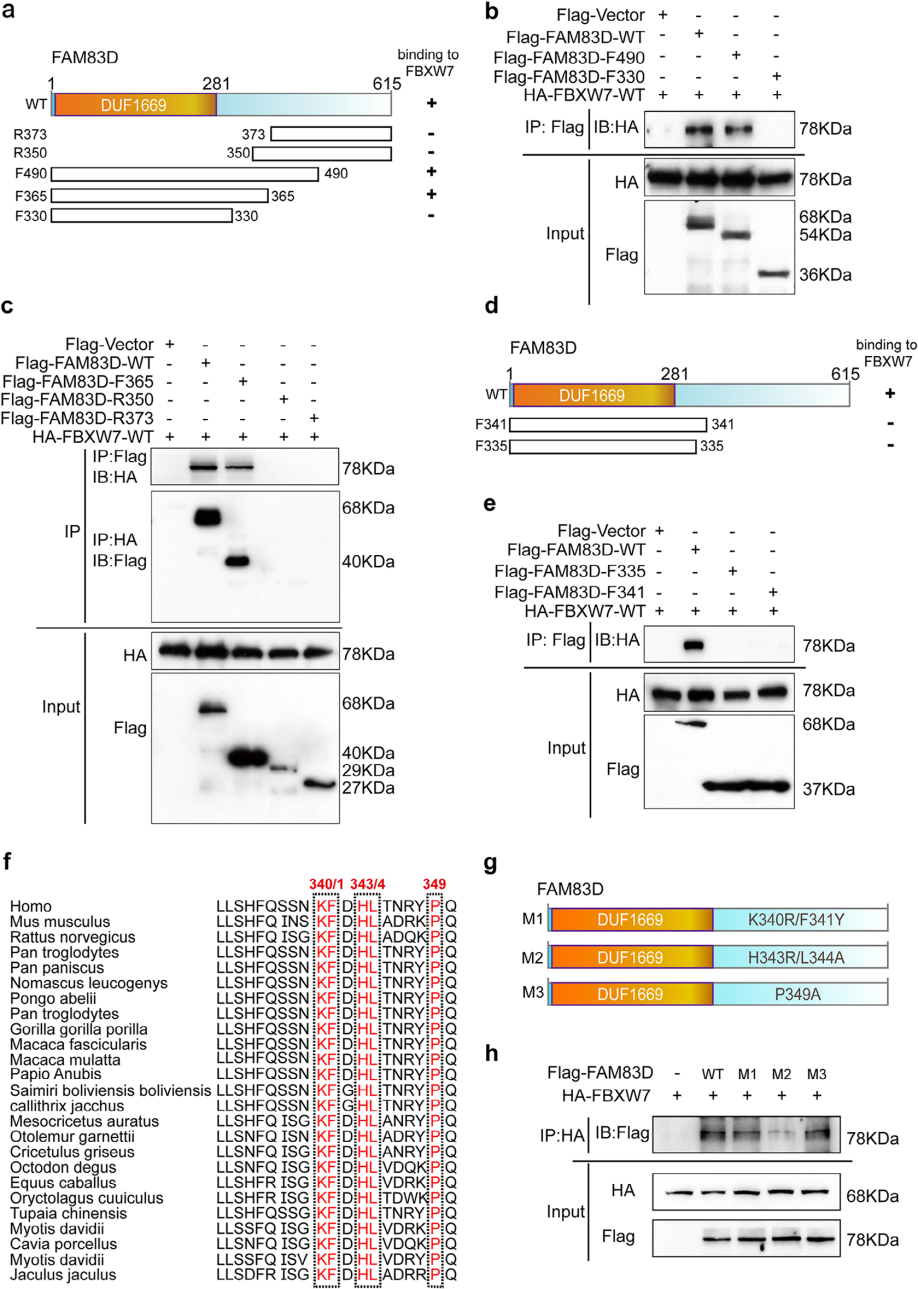
Identification of the FBXW7-binding sites on FAM83D. **a**, Schematic diagram of wild type (WT FAM83D) protein structure and its truncations (R373, R350, F490, F365 and F330). **b**-**c**, Co-immunoprecipitation analysis of the interaction between FBXW7 and FAM83D truncation mutants: F490 and F330 (**b**), F365, R350 and R373 (**c**). **d**, Schematic diagram of WT FAM83D protein structure and the refined truncations (the residues 330 to 350). **e**, Determination of FAM83D fragments (F335 and F341) for FBXW7 binding through co-immunoprecipitation analysis. **f**, Sequence alignment of residues from the indicated 25 different species using UniProt according to the regions 341 to 450 of human FAM83D potentially involved in FBXW7 binding. **g**, Schematic diagram of FAM83D point mutants (M1-M3). **h**, Identification of the key residues for FBXW7 binding by co-immunoprecipitation analysis. The results were the representative of three independent experiments

We previously discovered that FAM83D can bind to FBXW7 physically and down-regulate the protein level of FBXW7 [22]. To elucidate the importance of the FAM83D/FBXW7 interaction, we identified the structural region(s) of FAM83D that are responsible for FBXW7 binding through truncation analysis. Five truncations of FAM83D were first generated including F330 (1-330), F365 (1-365), F490 (1-490), R350 (350–615), and R373 (373–615) (Fig. 1a). HEK293T cells were co-transfected with Flag-tagged FAM83D truncations and HA-tagged FBXW7 respectively. Immunoprecipitation with anti-Flag antibodies and subsequent immunoblotting with anti-HA antibodies showed that both the full length FAM83D (WT) and the truncated F490 co-precipitated with FBXW7 while the truncated F330 failed to bind to FBXW7 (Fig. 1b). Moreover, co-immunoprecipitation (co-IP) assay revealed that the truncated R350 and R373 did not interact with FBXW7 in a reciprocal fashion (Fig. 1c). These results suggest that the residues 330 to 350 of FAM83D are required for binding to FBXW7. To further narrow down the interaction regions of FAM83D with FBXW7, we constructed another two truncations, F335 (1-335) and F341 (1-341) (Fig. 1d). Co-IP analysis showed that although FAM83D WT could precipitate with FBXW7, neither F335 nor F341 could bind to FBXW7, highlighting the importance of fragments from 341 to 350 for FBXW7 binding (Fig. 1e). Then, to better understand the key residues involved in FAM83D and FBXW7 interaction in these fragments (341–350), we performed sequence alignment from different species using UniProt and found that the amino acids K340/F341, H343/L344 and P349 were highly conserved in each species (Fig. 1f). Accordingly, we respectively generated three FAM83D mutants: double K340R/F341Y mutant (M1), double H343R/L344A mutant (M2) and a P349A mutant (M3) (Fig. 1g). Subsequent co-IP analysis revealed that FAM83D M1 and M3 mutants had similar strength of interaction with FBXW7 as the FAM83D WT (Fig. 1h). Nevertheless, FAM83D M2 exhibited remarkable reduction in binding to FBXW7 (Fig. 1h). Together, these results suggest that the residues 340 to 350 of FAM83D, especially amino acids H343 and L344 are involved in the interaction with FBXW7.

### FAM83D facilitates ubiquitination and degradation of FBXW7 in a H343/L344 dependent manner

**Fig. 2:**
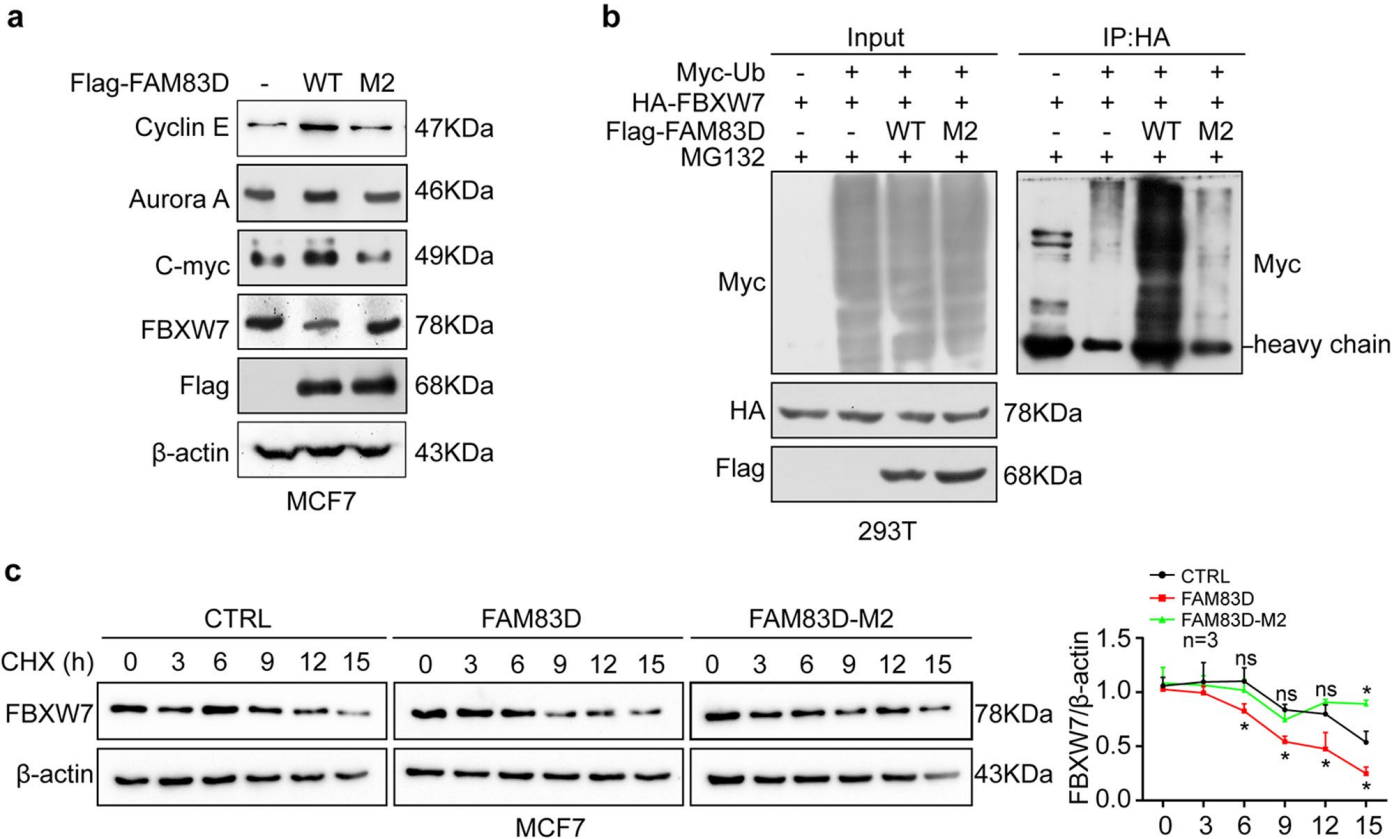
The importance of residues H343/L344 of FAM83D binding to FBXW7 for FAM83D-mediated down-regulation of FBXW7. **A**, The protein levels of FBXW7 and its downstream substrates including Cyclin E, Aurora A and c-Myc were detected by western blotting after FAM83D WT and H343R/L344A mutant (M2) were reintroduced into MCF7 cells. **B**, The effect of FAM83D H343R/L344A mutant (M2) on the ubiquitnation of FBXW7 was determined by co-immunoprecipitation analysis. HA-tagged FBXW7 was co-transfected into HEK293T cells with Myc-tagged ubiquitin (My-Ub) and Flag-tagged wild-type FAM83D (Flag-FAM83D WT), or Flag-tagged FAM83D mutant (Flag-FAM83D M2). Immunoprecipitation with anti-HA antibody followed by anti-Myc antibody immunoblotting. **C**, The effect of FAM83D H343R/L344A mutant (M2) on FBXW7 protein stability was determined by cycloheximide (CHX) chase assay. MCF7 cells were respectively transfected with FAM83D WT or H343R/L344A mutant (M2) followed by treatment with 50 µg/mL CHX for the indicated time intervals. Endogenous FBXW7 protein degradation was detected by western blotting. The graphs show quantitative analysis of CHX chase data. Data were presented by mean ± SD of three independent experiments. Ns: not significant. *: p < 0.05 based on the Student’s *t*-test

Since amino acids H343 and L344 were identified as the key sites that mediated FAM83D/FBXW7 interaction, we then questioned whether these sites are crucial for FAM83D-induced FBXW7 reduction. We reintroduced FAM83D WT and H343R/L344A mutant (M2) into MCF7 cells with low expression of endogenous FAM83D. The expression levels of FBXW7 and its downstream substrates were first evaluated by western blotting analysis. As previously shown, ectopic expression of FAM83D WT significantly inhibited FBXW7 expression accompanied by elevated levels of FBXW7 substrate proteins, including Cyclin E, Aurora A and c-Myc (Fig. 2a). FAM83D M2 overexpression, however, had no significant effect on the protein levels of FBXW7 and its downstream substrates (Fig. 2a). In our previous study, we found that the proteasome inhibitor MG-132 could ameliorate the FAM83D-triggered FBXW7 deficiency, indicating a regulatory potential of FAM83D on FBXW7 degradation. Thus, we further investigated the effect of FAM83D on the ubiquitination status and protein stability of FBXW7. HA-tagged FBXW7 was transfected into HEK293T cells with Myc-tagged ubiquitin (My-Ub) and Flag-tagged wild-type FAM83D (Flag-FAM83D WT), or Flag-tagged FAM83D mutant (Flag-FAM83D M2). Immunoprecipitation with anti-HA antibody followed by anti-Myc antibody immunoblottingting demonstrated that overexpression of FAM83D WT led to a significant increase of ubiquitination of FBXW7 whereas FAM83D M2 lost the ability to promote ubiquitination of FBXW7 (Fig. 2b). Likewise, cycloheximide (CHX) chase assay showed that FAM83D WT, but not FAM83D M2, greatly accelerated the turnover of FBXW7 (Fig. 2c). These data suggest that FAM83D negatively regulates FBXW7 levels by promoting its proteasomal degradation and the residues H343/L344 are required for FAM83D-mediated down- regulation of FBXW7.

### Mutation of H343/L344 impaired the oncogenic roles of FAM83D in BC

**Fig. 3:**
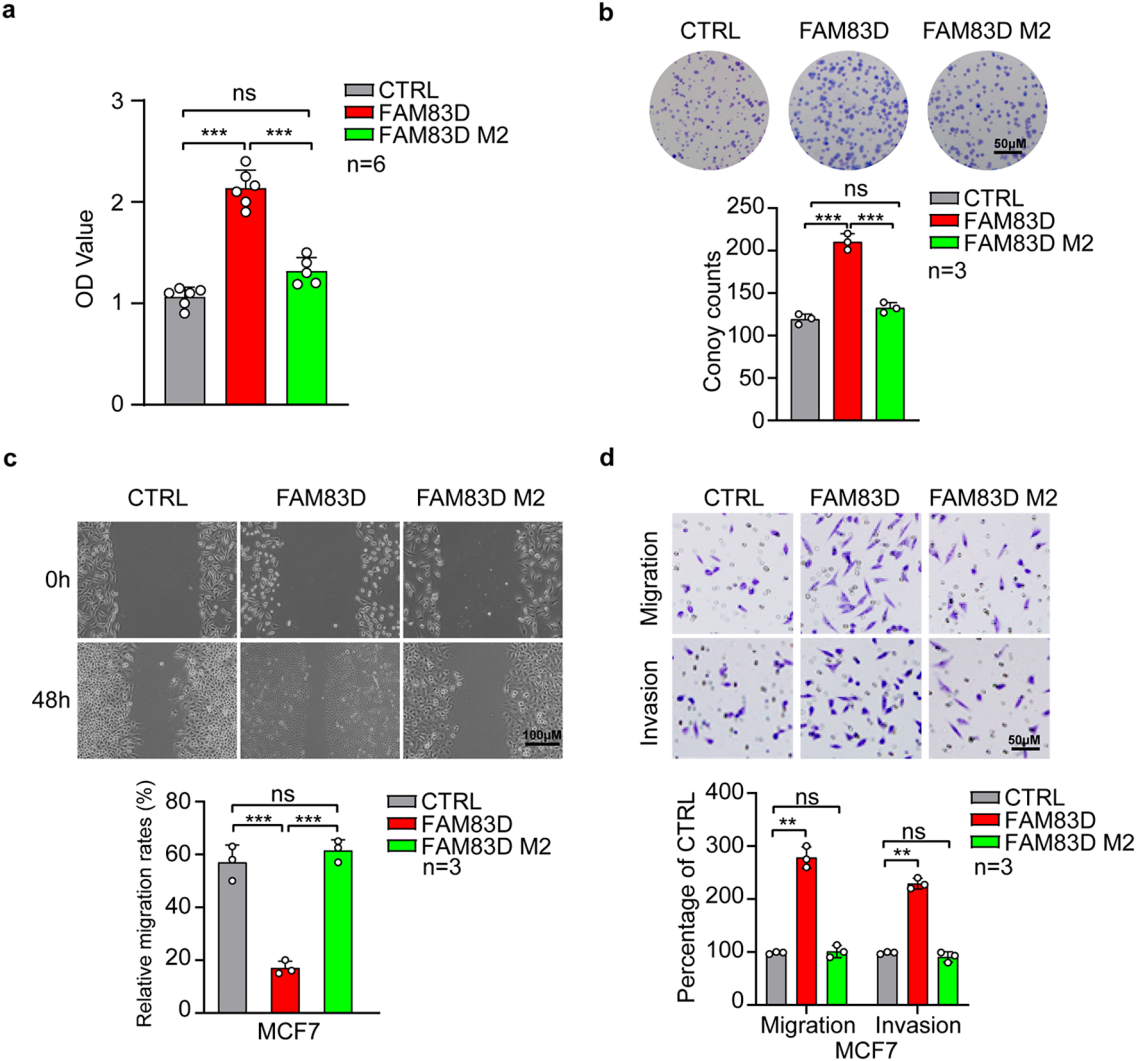
Evaluation of the significance of residues H343/L344 for the oncogenic roles of FAM83D in vitro. **a**, The effects of H343/L344 mutation (FAM83D M2) on the BC cell viability were determined by CCK8 assay. **b**, The effects of H343/L344 mutation (FAM83D M2) on the BC cell proliferation were detected by clonogenic assay. Quantitative analyses were shown in the graphs. **c**-**d**, The effects of H343/L344 mutation (FAM83D M2) on the BC cell migration and invasion were determined by wound-healing assay (**c**) and Matrigel coated or uncoated Transwell assay (**d**). Quantitative analyses were shown in the graphs. Data were presented as mean ± SD. ns: not significant. **: p < 0.01, ***: p < 0.001 based on the Student’s *t*-test

Given that mutation of H343/L344 abolished the regulation of FAM83D on FBXW7 expression, we next explored the significance of these binding sites in the cell proliferation and motility phenotypes induced by FAM83D. The influence of FAM83D M2 on the cell proliferation was monitored by Cell Counting Kit-8 (CCK-8) and clonogenic assays. We found that contrary to the strong growth promoting activity of FAM83D WT, augmented expression of FAM83D M2 didn’t affect cell viability as well as clonogenic activity dramatically (Fig. 3a, b). Similarly, scratch healing assay and Boyden chamber migration/invasion assay showed that FAM83D WT largely increased the ability of cell migration and invasion but FAM83D M2 failed to promote such capacity of BC cells (Fig. 3c, d).

**Fig. 4:**
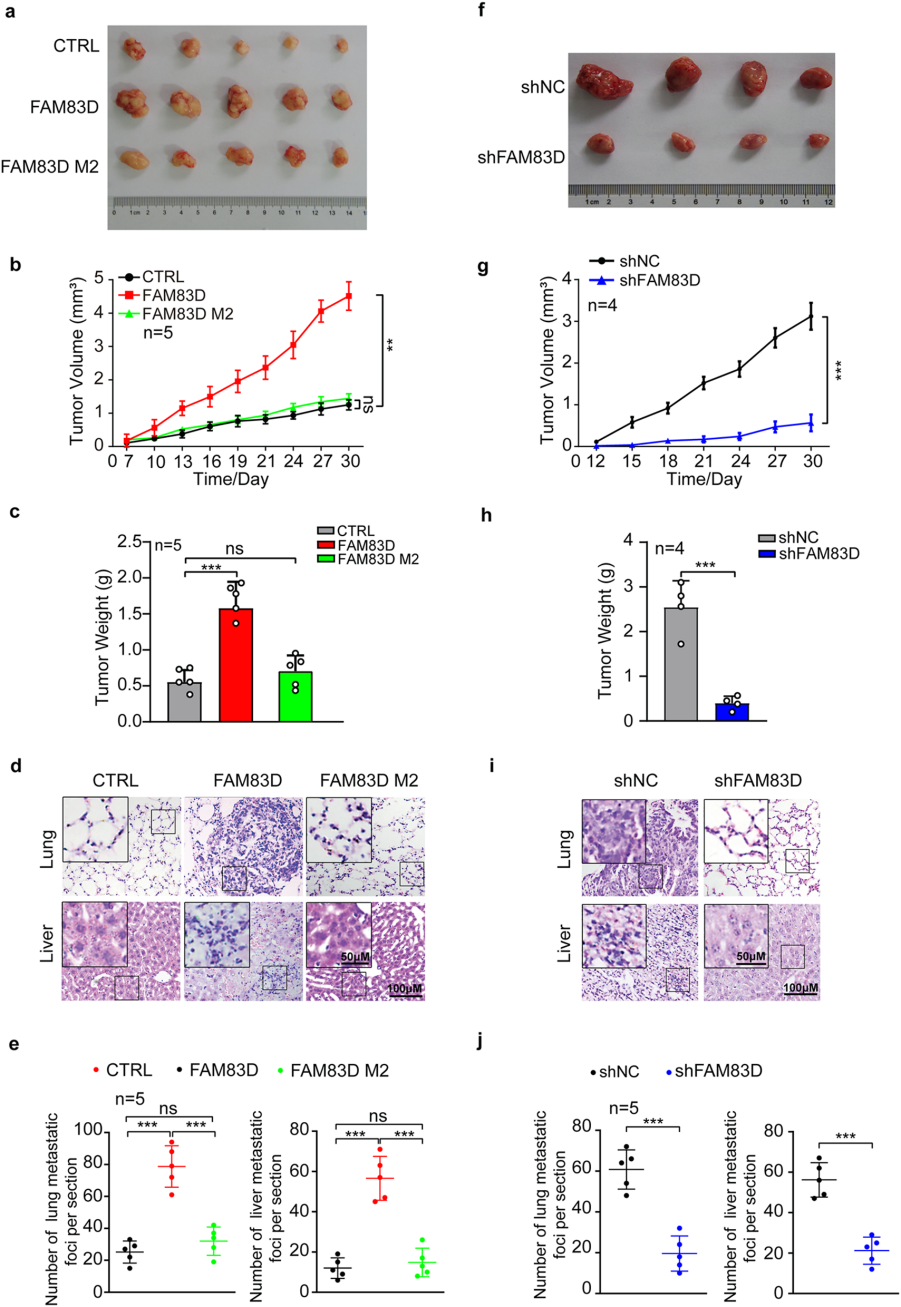
Determination of the significance of residues H343/L344 for the oncogenic roles of FAM83D in vivo. **a**-**c**, The empty vector control (CTRL), FAM83D WT-overexpressed (FAM83D WT), and FAM83D M2-overexpressed (FAM83D M2) MCF7 cells were subcutaneously inoculated in the nude mice (n = 5/group). Representative images of the dissected tumors. A ruler was used to demonstrate the size of the tumors (**a**). The tumor growth curve of each group was generated by measuring every 3 days (**b**). Quantification of tumor weights at the end point (**c**). **d**-**e**, The aforementioned cells were injected into the tail vein of nude mice (n = 5/group). Representative hematoxylin and eosin staining of metastatic foci per section in lung (d, upper) and liver (d, lower) of individual mouse. Quantification of the metastatic nodules per section in lung (e, left) and liver (e, right). **f-h**, FAM83D-silenced BT549 (shFAM83D) and their control cells (shNC) were subcutaneously inoculated in the nude mice (n = 4/group). Representative images of the dissected tumors. A ruler was used to demonstrate the size of the tumors (**f**). The tumor growth curve of each group was generated by measuring every 3 days (**g**). Quantification of tumor weights at the end point (**h**). **i-j**, The indicated BT-549 cells were injected into the tail vein of nude mice (n = 4/group). Representative hematoxylin and eosin staining of metastatic foci per section in lung (i, upper) and liver (i, lower) of individual mouse. Quantification of the metastatic nodules per section in lung (j, left) and liver (j, right). Data were presented as mean ± SD. ns: not significant. **: p < 0.01, ***: p < 0.001 based on the Student’s *t*-test

Further, to validate such findings in vivo, we subcutaneously inoculated the empty vector control (CTRL), FAM83D WT-overexpressed (FAM83D WT), and FAM83D M2-overexpressed (FAM83D M2) MCF7 cells in the athymic nude mice, and routinely monitored the tumor growth. We observed that FAM83D WT overexpression markedly promoted tumor growth, but FAM83D M2 had no significant effect on the tumor growth, as indicated by representative images, tumor weight and growth curve (Fig. 4a-c). In the meantime, we also injected aforementioned cells into nude mice through the tail vein. After forty days, the mice were sacrificed and the liver and lung of the mice were collected for hematoxylin and eosin (HE) staining to assess the metastasis status. In line with the observations in vitro, FAM83D WT dramatically increased the number of metastatic tumors in both lung and liver of each mouse while FAM83D M2 lost the pro-metastatic activity evidenced by the similar number of metastatic nodules in both lung and liver with the control group (Fig. 4d, e). Finally, knockdown of FAM83D in BT549 significantly inhibited tumor growth (Fig. 4f-h) and metastasis (Fig. 4i, j) in vivo. Collectively, these results indicate that FAM83D is an oncogene and mutation of H343/L344 impaired FAM83D-induced oncogenic phenotypes both in vitro and in vivo.

### FAM83D exerted oncogenic roles through suppressing FBXW7

**Fig. 5:**
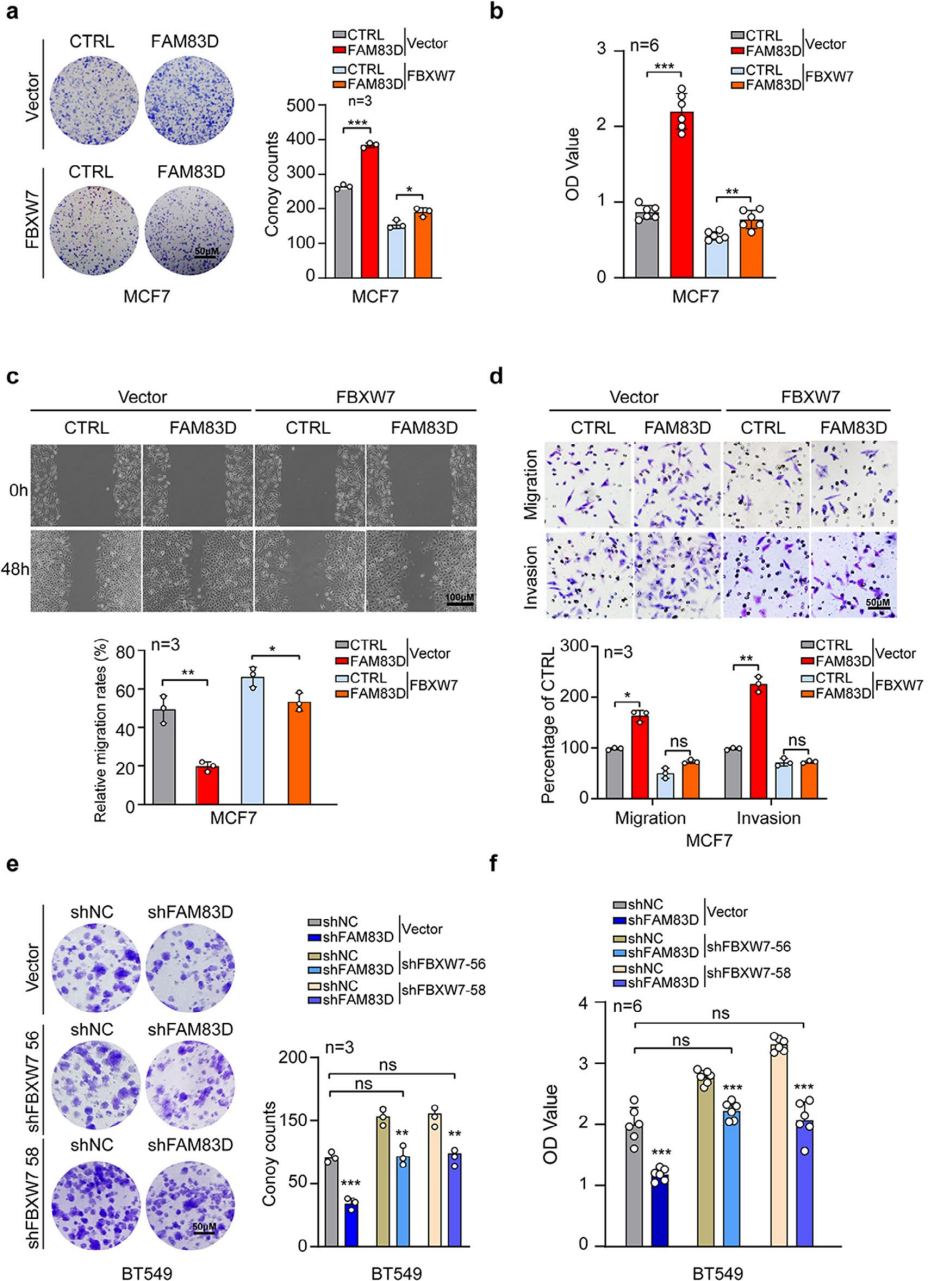
FBXW7 deficiency mediates the oncogenic roles of FAM83D. **a-d**, FBXW7 expression plasmid or empty vector was introduced into FAM83D-overexpressed MCF7 cells. The effects of FBXW7 re-expression on FAM83D-induced cell proliferation were detected by clonogenic assay (**a**) and CCK8 assay (**b**). The effects of FBXW7 re-expression on FAM83D-induced cell invasiveness and migration were determined by wound-healing assay (**c**) and Matrigel coated or uncoated Transwell assay (**d**). **e-f**, FBXW7-specific shRNA (shFBXW7 56 and shFBXW7 58) or control shRNA was introduced into FAM83D-silenced BT-549 cells. The effects of FBXW7 depletion on FAM83D loss-induced cell proliferation inhibition were examined by clonogenic assay (**e**) and CCK8 assay (**f**). Graphs in a, c, d and e presented quantification analyses. Data were presented as mean ± SD. ns: not significant. *: p < 0.05, **: p < 0.01, ***: p < 0.001 based on the Student’s *t*-test

Considering the inhibition of FAM83D on FBXW7 expression and the well-recognized tumor suppressive roles of FBXW7 in multiple cancers [28–32], we then investigated whether FAM83D-induced malignant phenotypes of BC cells was mediated by FBXW7 down-regulation. We restored FBXW7 expression in FAM83D-overexpressed MCF7 cells through transfection of FBXW7 expression plasmids (Extended Data Fig. 1a). We found that forced expression of FBXW7 almost completely abolished the proliferation-promotive effect of FAM83D on MCF7 cells, as indicated by clonogenic and CCK-8 assays (Fig. 5a, b). Moreover, overexpression of FBXW7 significantly alleviated the increase of cell migration and invasion triggered by augmented expression of FAM83D in MCF7 cells (Fig. 5c, d). Then, we knocked down FBXW7 in FAM83D-silenced BT549 and MDA-MB-231 cells to restore FBXW7 expression to the near-basal levels (Extended Data Fig. 1b, c). Strikingly, FBXW7 silencing nearly reversed the inhibitory effects of FAM83D deficiency on these cells’ growth, migration and invasion (Fig. 5e, f, Extended Data Fig. 2a, b, Extended Data Fig. 3a-d). Together, these data suggest that FBXW7 mediates the impact of FAM83D on cell proliferation and motility phenotypes of BC cells.

### FAM83D negatively correlates with expression of FBXW7 in BC tissues

**Fig. 6:**
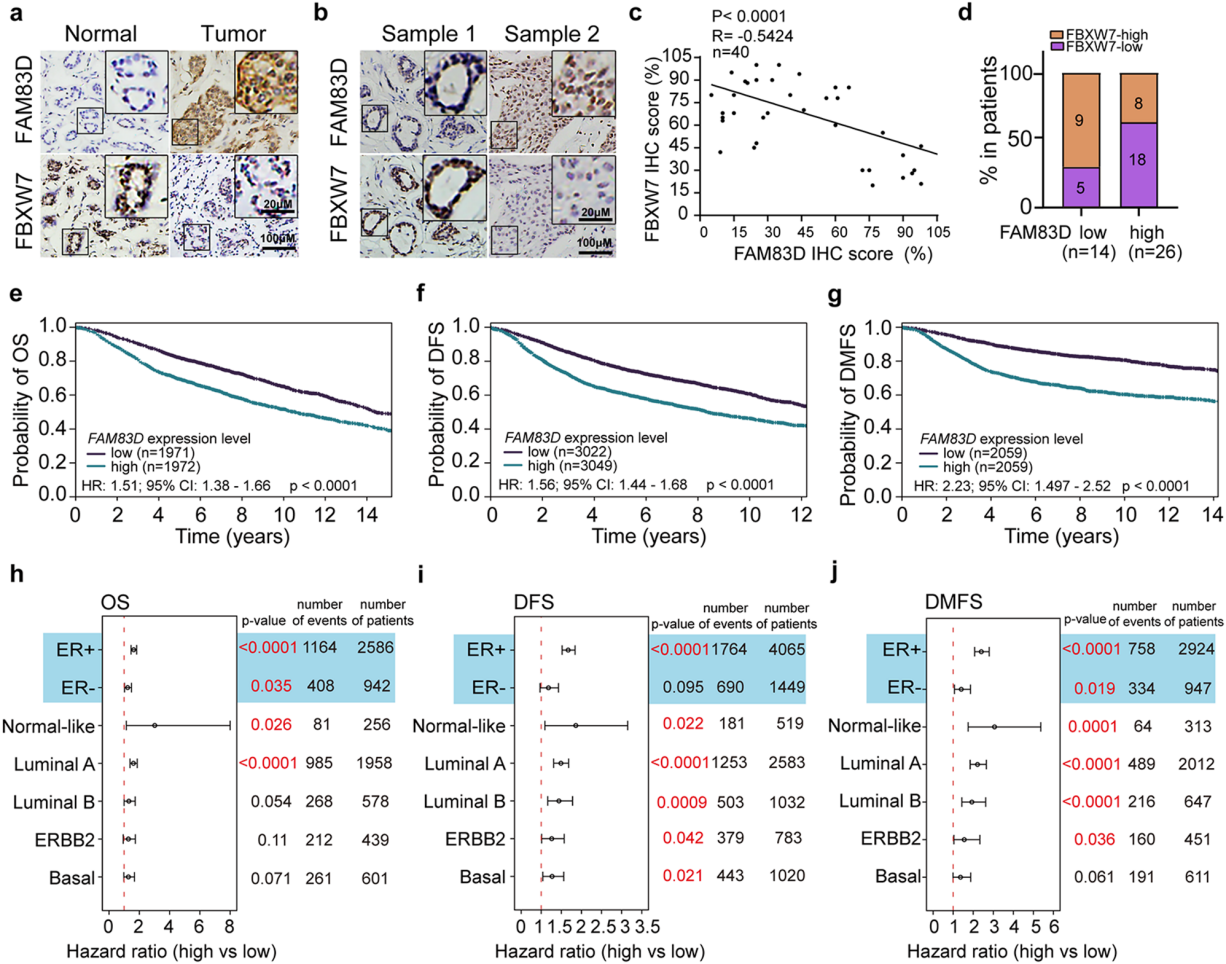
FAM83D expression negatively correlates with FBXW7 expression in BC tissues and positively associated with poor prognosis of patients with BC. **a**, Representative images of IHC staining for FAM83D and FBXW7 protein in paired breast cancer (BC) tissues and the non-cancerous tissues. **b**, Representative images of IHC staining for FAM83D and FBXW7 protein in BC tissues. **c**, The correlation between the expression of USP35 FAM83D and FBXW7 in BC tissues (n = 40). **d**, The box plot indicated the relative FBXW7 level in FAM83D-low and FAM83D-high patients (Median FAM83D or FVXW7 expression was defined as cut-off point). **e-g**, Significant association of FAM83D expression with overall survival (OS) (**e**), disease-free survival (DFS) (**f**), and distant metastasis-free survival (DMFS) (**g**). The Kaplan-Meier curves were generated using Breast Cancer Gene-Expression Miner v5.0. **h-j**, The forest plot shows the association of FAM83D expression with overall OS (**h**), DFS (**i**), and DMFS (**j**) in different ER status and PAM50 molecular subtypes. All p-values were obtained from the log-rank test

Finally, to evaluate the clinical relevance of FAM83D-regulated FBXW7 expression, we examined FAM83D and FBXW7 protein expression in BC patient samples through immunohistochemistry (IHC) staining. Consistent with the previous findings, the FAM83D expression was higher in the breast cancer tissue compared to the non-cancerous tissues, but the expression of FBXW7 is lower in breast cancer tissues than that in adjacent tissues (Fig. 6a). Specifically, FBXW7 was highly increased in the samples with low FAM83D expression but decreased in those with relatively high FAM83D expression (Fig. 6b). Quantification and linear regression analyses revealed that FBXW7 level was negatively correlated with FAM83D expression, further confirming the negative regulation of FAM83D on FBXW7 expression (Fig. 6c, d).

### Elevated expression of FAM83D confers poor prognosis and resistance to cancer therapy in BC

**Fig. 7:**
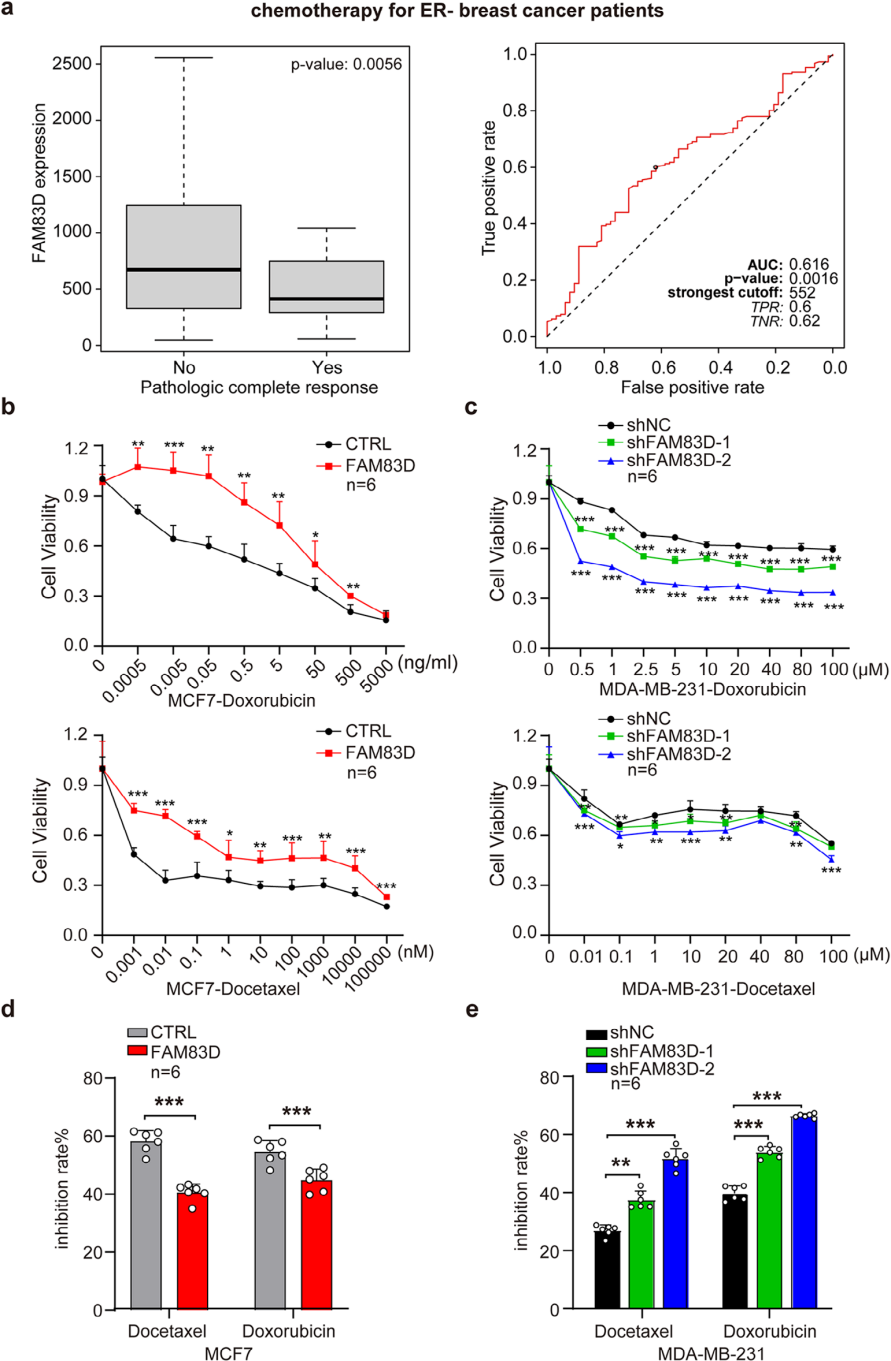
FAM83D promotes chemo-resistance in BC. **a**, FAM83D expression significantly correlated with pathological complete response (pCR) to chemotherapy in ER- breast cancer patients. Significant difference in FAM83D expression between two response groups (left panel) and the predictive value of FAM83D expression for pCR to chemotherapy (right panel) were obtained ROC plotter (https://www.rocplot.org/). **b-c**, The FAM83D-overexpressed MCF7 cells (b) or FAM83D-silenced MDA-MB-231 cells (c) and their control cells were treated with different concentrations of Doxorubicin (upper), or Docetaxel (lower) for 48 h (n = 6). The cell viability was assessed using CCK8 assay. **d**, The inhibition rate of 0.5 µg/mL Doxorubicin or 100 nM Docetaxel on FAM83D-overexpressed MCF7 cells and their control cells (n = 6). **e**, The inhibition rate of 10 µM Doxorubicin, or 20 µM Docetaxel on FAM83D-silenced MDA-MB-231 cells and its control cells (n = 6). Data were presented as mean ± SD. ns: not significant. **: p < 0.01, ***: p < 0.001 based on the Student’s *t*-test

Using Breast Cancer Gene-Expression Miner v5.0 (bc-GenExMiner v5.0), we conducted a meta-evaluation of clinical significance of FAM83D in BC and found that high expression levels of FAM83D are significantly associated with poor prognosis regarding to overall, disease-free, and distant metastasis-free survival (Fig. 6e-g). The prognostic value of FAM83D is independent of ER status and PAM50 molecular subtypes (Fig. 6h-j). Furthermore, we found patients with high transcriptional level of FAM83D confer resistance to endocrine therapy (Extended Data Fig. 4) and to chemotherapy (Fig. 7a) using ROC plotter (https://www.rocplot.org/). Consistent with this observation, ectopic overexpression of FAM83D in MCF7 cells makes them resistant to doxorubicin and docetaxel treatment (Fig. 7b), while knockdown of FAM83D in MDA-MB-231 cells makes them sensitive to these treatments (Fig. 7c). Taken together, these data indicated that elevated expression of FAM83D confers poor prognosis and resistance to cancer therapy in BC.

## Discussion

According to the 2022 Global Cancer Statistics, BC is the most common malignant tumor in women with 31% incidence and 15% mortality, which is one of the most serious dangers to women worldwide [1]. Despite improvements in clinical diagnosis and therapeutic strategies, the prognosis of a substantial portion of BC patients is still gloomy partially due to the inherent molecular heterogeneity which dictates the distinct therapeutic responses [3, 33, 34]. Thus, defining the molecular landscape in BC and unraveling novel contributors to BC initiation and progression is of great significance for discovering effective drug targets to improve treatments and prognosis for the BC patients. FAM83D is the newly identified member of FAM83 (Family with sequence similarity 83) protein family which has 8 members consisting of FAM83A, FAM83B, FAM83C, FAM83D, FAM83E, FAM83F and FAM83G. *FAM83D* gene is located on chromosome 20q which is frequently amplified thus resulting in high expression in the majority of human cancer types including ovarian cancer, colorectal cancer, gastric cancer, esophageal cancer, lung cancer, breast cancer, hepatocellular carcinoma and pancreatic adenocarcinoma [11, 13, 16–20, 22, 35, 36]. Over-expressed FAM83D promotes neoplastic transformation and positively correlates to aggressive tumor biology, high-grade tumors, and poor prognosis in a variety of cancers [13, 16, 20, 37, 38]. It has been reported that FAM83D exerts its oncogenic roles through activating critical signaling pathways. For instance, FAM83D promotes cell invasion and chemo-resistance by regulating AKT/mTOR and TGFβ1-pSMAD2/3 signaling in lung and ovarian cancer [16, 39, 40]. FAM83D enhances cell proliferation by activating the MEK/ERK signaling pathway in hepatocellular carcinoma [41]. Moreover, FAM83D stimulates cell proliferation and motility through the Wnt/β-catenin pathway in gastric cancer and pancreatic adenocarcinoma [11, 38]. Interestingly, we and other two groups have identified that FAM83D could negatively regulate the protein level of FBXW7, a well-recognized tumor suppressor, and thereby accelerating the carcinogenesis in breast, hepatic and colorectal cancers, suggesting that FAM83D-mediated FBXW7 downregulation was an important mechanism underlying the oncogenic action of FAM83D [19, 22, 42]. However, till now, how FAM83D interacts with FBXW7 and inhibits its expression remains unknown.

In this study, we first investigated the molecular basis of FAM83D interaction with FBXW7 and identified two critical amino acids, H343 and L344, on FAM83D, which are required for FBXW7 binding through a comprehensive mutational analysis and subsequent Co-IP analysis. Functional analysis further revealed that H343/L344 double mutations failed to decrease FBXW7 expression and remarkably ameliorated the tumor-promotive effects of FAM83D both in vitro and in vivo, highlighting the importance of these sites in biological activities of FAM83D. Since we and other have shown that overexpression of FAM83D confers poor prognosis and resistance to chemotherapy and other cancer treatments [16, 37], future studies may shed more lights on whether and how targeting the FBXW7-binding sites on FAM83D will have good curative effect against multiple cancer types.

As a well-known tumor suppressor, FBXW7 is frequently inactivated or loss of expression in a wide array of human cancers. Growing number of studies have shown that multiple mechanisms contributed to the deficiency of FBXW7 in cancers including post-transcriptional regulation at protein level such as phosphorylation-induced mislocalization or loss of dimerization thereby encouraging self-ubiquitination, and cis- or trans-ubiquitination triggered by imbalanced specific E3 ligases and deubiquitinating enzymes followed by accelerated degradation. Our present study has further demonstrated that FAM83D could promote the ubiquitination and subsequent proteasomal degradation of FBXW7 thus decreasing FBXW7 protein level in an H343/L344 dependent manner. How FAM83D promotes the ubiquitination of FBXW7 requires further investigation. Nevertheless, our findings provide strong evidence that FAM83D post-translationally regulates FBXW7.

Although we have identified FBXW7 as a downstream target of FAM83D, there is still lacking direct evidence linking FBXW7 to the oncogenic roles of FAM83D in BC. Here, through re-introduction of FBXW7 in FAM83D overexpressed MCF7 cells or knockdown of FBXW7 in FAM83D silenced BT549 cells, we found that augmented FBXW7 ameliorated the stimulative effects of FAM83D on cell proliferation, migration and invasion whereas FBXW7 ablation nearly reversed the anti-tumor phenotype induced by FAM83D deficiency, indicating that FBXW7 mediates the function of FAM83D on cell growth and mobility in BC. Moreover, the negative correlation between FAM83D protein and FBXW7 protein was further verified in the clinical samples. Interaction between FAM83D and FBXW7 plays an important role in cancer development.

In summary, this study revealed that the H343 and L344 residues of FAM83D were critical for its regulation on FBXW7 as well as its oncogenic roles in BC. We have also defined the mechanism underlying the FAM83D down-regulation on FBXW7 protein and preliminarily explored the biological and clinical significance of such regulation in progression and prognosis of patients with BC. Our finding suggests that FAM83D-FBXW7 axis is a potential player in promoting the malignant transformation and affecting the chemotherapy response. Blocking FAM83D-FBXW7 interaction may provide an attractive therapeutic strategy for BC and other cancer patients.

## Methods

### Cell culture and transfection

HEK293T and BC cell lines (MCF7, MDA-MB-231 and BT549) were purchased from the Cell Bank of the Chinese Academy of Science (Shanghai, China). MCF7, MDA-MB-231 and HEK293T cells were cultured in DMEM medium with 10% fetal bovine serum (FBS). BT549 cells were cultured in RPMI-1640 medium with 10% FBS. All the cell lines were cultured at 37 °C in a 5% CO2/95% air atmosphere and were revived every 3 to 4 months. For all transfection procedures, standard protocols were followed by the manufacturer’s instructions using Lipofectamine 2000 (Invitrogen, Waltham, MA, USA).

### Expression plasmids and RNA interference

Human wild-type FAM83D (pCMV-3X-Flag-FAM83D), FBXW7 (pcDNA3.1-HA-FBXW7), Ubiquitin (pCMV-3X-Myc-Ub) expression vectors and the lentiviral constructs expressing human FBXW7 short hairpin RNA (shFBXW7-56/58) were previously constructed by our laboratory [22, 43]^,^ [44]. A set of FAM83D truncations was generated by subcloning the polymerase chain reaction (PCR) products into the pCMV-3X-Flag vectors digested by EcoR1 and BamH1. The point mutant plasmids of FAM83D were generated by Quikchange mutagenesis kit (Stratagene, CA, USA). All primers were synthesized by Boshang Biotechnology Co. (Jinan, China) and Qingke Biotechnology Co. (Beijing, China). The primer sequences were listed in Extended Data Table 1.

The plasmid psPAX2 and pM’2.G (GeneChem Co., Shanghai, China) were used for lentiviral packaging. Human FAM83D cDNA was cloned into the pLVX-IRES-Puro vector (Addgene, MA, USA) and the specific shRNAs targeting FAM83D were cloned into the pLKO.1-TRC vector (GenePharma, Shanghai, China) (shRNA1: CCTGACTTTGTCACCTTTGTT and shRNA2: GATCTGAAAGTTCATCCTGAA, designated shFAM83D-1 and shFAM83D-2). Empty vector or scrambled shRNA (nonspecific sequence TTCTCCGAACGTGTCACGTTT, designated as scramble) were used as corresponding controls.

### Antibodies and reagents

The antibodies used in our experiments are listed in Extended Data Table 2. The protein synthesis inhibitor, cycloheximide (CHX), the protease inhibitor MG132 and protein G/A magnetic bead were from Calbiochem (Darmstadt, Germany). The chemotherapeutic drugs Docetaxel and Doxorubicin were purchased from Beyotime Biotechnology (Shanghai, China).

### Western blotting and co-immunoprecipitation

Western blotting (WB) and co-immunoprecipitation (co-IP) were performed as previously described [45]. In brief, for Western blotting, 30 µg total protein extracted from the indicated BC cells using RIPA buffer (Thermo Fisher Scientific, MA, USA) was separated by SDS-PAGE followed by electrically transferred to polyvinylidene fluoride (PVDF) membrane (Millipore, MA, USA). Then the membrane was incubated with indicated primary and secondary antibodies. For co-IP analysis, 1 mg total protein in IP Lysis Buffer (Thermo Fisher Scientific, MA, USA) was first incubated with indicated primary antibody and purified by protein G/A magnetic bead (Calbiochem, Darmstadt, Germany). Then the co-immunoprecipitated interacting proteins were analyzed by SDS-PAGE and western blotting.

### Cell viability and Colony formation assay

The cell viability was measured by Cell Counting Kit-8 (CCK-8, APExBIO, HOU, USA) assay and clonogenic assay. For CCK-8 assay, as previously reported [46, 47], 2000 cells seeded in the 96-well plates with three repetitions were cultured for 48 h to examine the cell viability or cultured for 24 h followed by treatment with the indicated drugs for further 48 h at different concentrations to examine the drug toxicity. The absorbance at 450 nm of each well was measured 4 h after addition of 10 µL CCK8 solution to each well of the plate. For clonogenic assay, 1 × 10^3^ cells per well were seeded into 6-well plates with three repetitions. After 12-day incubation, cells were fixed with methyl alcohol for 30 min and stained with Giemsa staining solution (Sigma, MO, USA).

### Wound-healing assay and transwell migration and invasion assay

For Wound-healing assay, MCF7, MDA-MB-231 and BT549 cells were cultured in 6-well plates. When the cell reached 100% confluence, linear wound was generated using a 200 µL pipette tip. After the cells were washed three times, photographs were taken immediately with phase contrast microscopy. Then, the cells were cultured in serum-free medium for another 48 h and the wound healing was determined at the same location. For Transwell migration and invasion assay, 2.5 × 10^4^ cells were seeded into the upper chamber of Transwell chamber (Corning, NY, USA) uncoated or coated with Matrigel (Corning, NY, USA). After 24 h of cultivation, they were fixed and stained with crystal violet. Migrated or invaded BC cells were counted in 10 randomly selected fields at ×100 magnification under an inverted light microscope.

### Subcutaneous and metastatic xenograft model

5-week-old female BALB/c nude mice were purchased from Vital River Laboratory Animal Technology Co. (Beijing, China). The animals were bred in specific-pathogen-free conditions with a 12-h light–dark cycle. The experiments were approved by the ethics committee of the School of Basic Medical Sciences, Shandong University and were performed in guidance with animal experiments in the Laboratory Animal Center of Shandong University. All mice were randomly divided into the subcutaneous injection group and tail vein injection group. For subcutaneous inoculation, the indicated tumor cells (1 × 10^6^) were resuspended in PBS medium and inoculated subcutaneously into the axilla of each mouse (n = 5 per group). The tumors were measured every 3 days after 7 days of inoculation and the tumor volume was calculated by the formula (length × width^2^)/2. The mice were killed 30 days after inoculation. For metastasis assays, cells were resuspended in 100 µL PBS at a concentration of 1 × 10^7^ cells/mL. Cell suspension (100 µL) was injected into tail veins of nude mice (n = 5 per group). All of the mice were killed 40 days after inoculation. When assuring the death of the mice, liver and lung tissues were extracted from the mice and were fixed in 4% paraformaldehyde followed by hematoxylin-eosin (HE) staining to count the number of metastatic nodules.

### Immunohistochemistry and scoring

40 human breast cancer tissues along with their adjacent noncancerous tissues were obtained from the tumor tissue bank from the Shandong Provincial Hospital (Shandong, China). Informed consent was obtained from the patients for this study and the study was approved by the ethics committee of the School of Basic Medical Sciences, Shandong University. The clinical samples embedded in paraffin were sliced into 5 μm sections. The immunohistochemistry (IHC) staining and the blind scoring were performed as previously described [48]. Briefly, the sections were incubated with FBXW7 (1:200, Abcam, Cambridge, UK) and FAM83D (1:100, Proteintech, Chicago, USA) antibodies. Staining was observed in 5 randomly selected high-power fields. The quantitation of IHC score was obtained by multiplying the weighted intensity (0 is no staining, 1 is weak staining, 2 is moderate staining and 3 is strong staining) and the percentage of positive cells (1 for less than 25%, 2 for 26–50%, 3 for 51–75%, and 4 for more than 75%). The scoring results were analyzed by two experienced pathologists.

### Statistical analysis

GraphPad Prism 8 software (La Jolla, CA, USA) was used for statistical analyses. The quantified data were presented as the mean ± SD of at least three independent experiments. Different groups were compared using unpaired, two-tailed, Student’s *t*-test. Spearman’s correlation analysis was used for analyzing the correlation between the expression of FBXW7 and FAM83D in the breast cancer samples. We performed a meta-analysis of the association between FAM83D expression and the overall survival (OS), disease-free survival (DFS), and distant metastasis-free survival (DMFS), generated Kaplan–Meier survival curve plots using Breast Cancer Gene-Expression Miner v5.0 (http://bcgenex.ico.unicancer.fr/BC-GEM/GEM-Accueil.php?js=1). Predictive value of FAM83D for responses to cancer treatments was assessed using ROC plotter (https://www.rocplot.org/). p < 0.05 was considered statistically significant.

### Electronic supplementary material

Below is the link to the electronic supplementary material.

Supplementary Material 1: Extended data file

Supplementary Material 2: Unprocessed western blots for different figures

## Data Availability

All data used in the association of FAM83D with clinical outcomes and responses to cancer treatments were downloaded from publicly available databases. All the data supporting the findings of this study are available from the corresponding author on reasonable request.
