# Supplementary material for: The FBXW7-binding sites on FAM83D are potential targets for cancer therapy
Source: Breast Cancer Res. 2024 Mar 7;26:37. doi: 10.1186/s13058-024-01795-9 (PMC10918900; doi:10.1186/s13058-024-01795-9)
Supplement: Supplementary file 1 — Supplementary Material 1: Extended data file [file 13058_2024_1795_MOESM1_ESM.docx]

**­­­­­Extended Data File for**

**The FBXW7-binding sites on FAM83D are potential target for cancer therapy**

Xiaoyu Jiang^1#^, Yuli Wang^1,2#^, Lulu Guo^1^, Yige Wang^1^, Tianshu Miao^1^, Lijuan Ma^1,3,^, Qin Wei^1^, Xiaoyan Lin^4^, Jian-Hua Mao^5^*, Pengju Zhang^1^*

**Authors’ affiliation**

^1^Key Laboratory Experimental Teratology of the Ministry of Education, Department of Biochemistry and Molecular Biology, School of Basic Medical Sciences, Cheeloo College of Medicine, Shandong University, Jinan, Shandong, 250012, China.

^2^Department of Clinical Laboratory, The Second Hospital of Shandong University, No. 247 Beiyuan Street, Jinan, Shandong, 250033, China.

^3^Department of Clinical Pharmacy, College of Pharmacy, Xinxiang Medical University, Xinxiang, Henan 453000, China.

^4^Department of Pathology, Shandong Provincial Hospital Affiliated to Shandong University, Jinan, China.

^5^Biological Systems and Engineering Division, Lawrence Berkeley National Laboratory, Berkeley, CA, USA.

^#^Equal contribution to this work

***Corresponding Authors:**

*E-mail addresses:* [jhmao@lbl.gov](mailto:jhmao@lbl.gov); [zhpj@sdu.edu.cn](mailto:zhpj@sdu.edu.cn)

**This Extended Data File includes:**

Extended Data Figures 1-4

Extended Data Tables 1-2

**
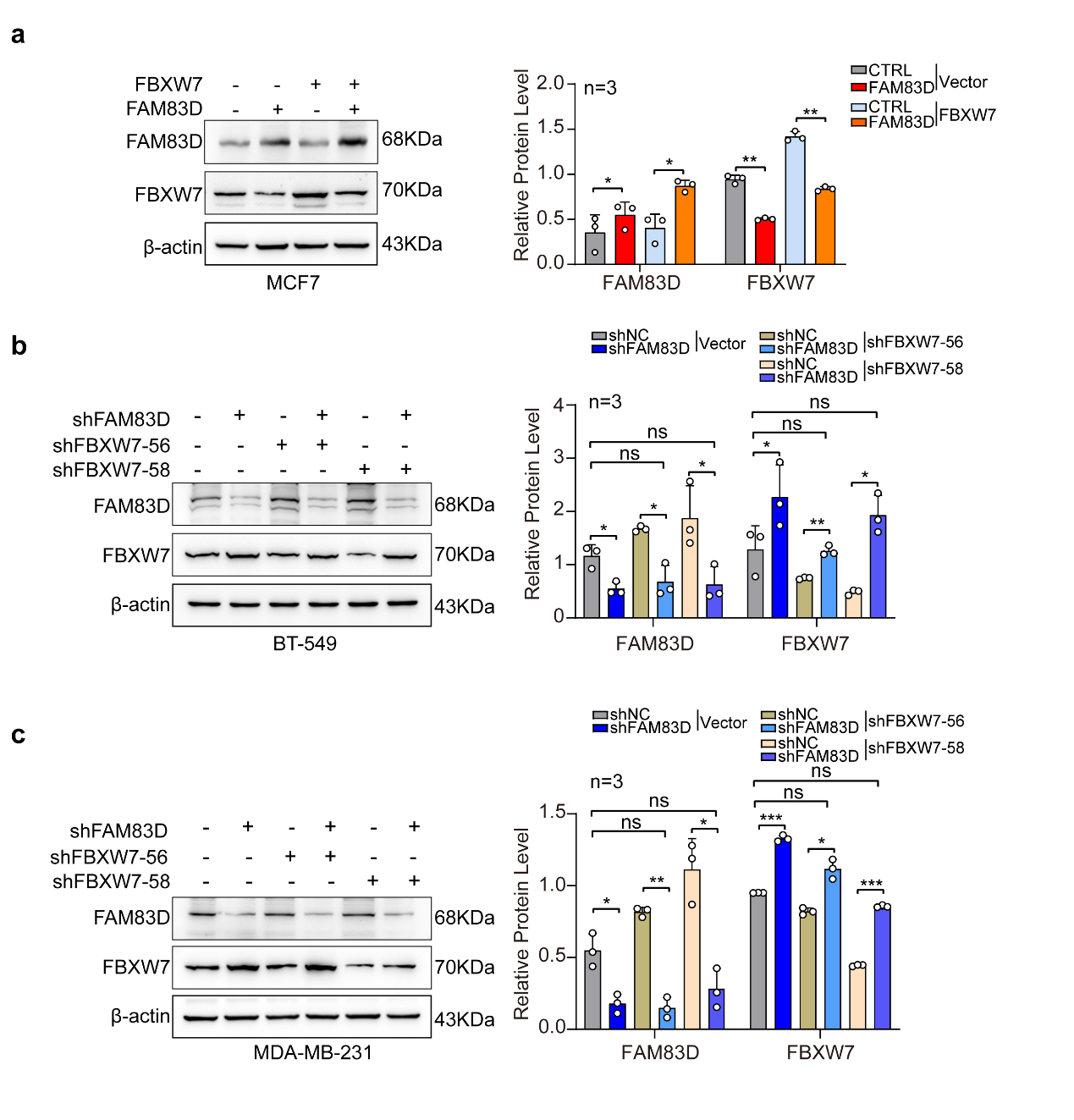
**

**Extended Data Fig. 1 The protein level of FBXW7 and FAM83D in the indicated cells. a,** The protein level of FBXW7 and FAM83D was determined by western blotting after FBXW7 was introduced into FAM83D-overexpressed MCF7 cells. Quantitative analyses were shown in the graphs (n=3). **b-c,** The protein level of FBXW7 and FAM83D was determined by western blotting after knockeding down FBXW7 in FAM83D-silenced BT549 (b) and MDA-MB-231 cells (c). Quantitative analyses were shown in the graphs (n=3). Data are presented by mean ±SD. ns: not significant, ^✱^P<0.05, ^✱✱^P<0.01, ^✱✱✱^P<0.001 based on the Student’s test. All results are representative of three independent experiments.

**
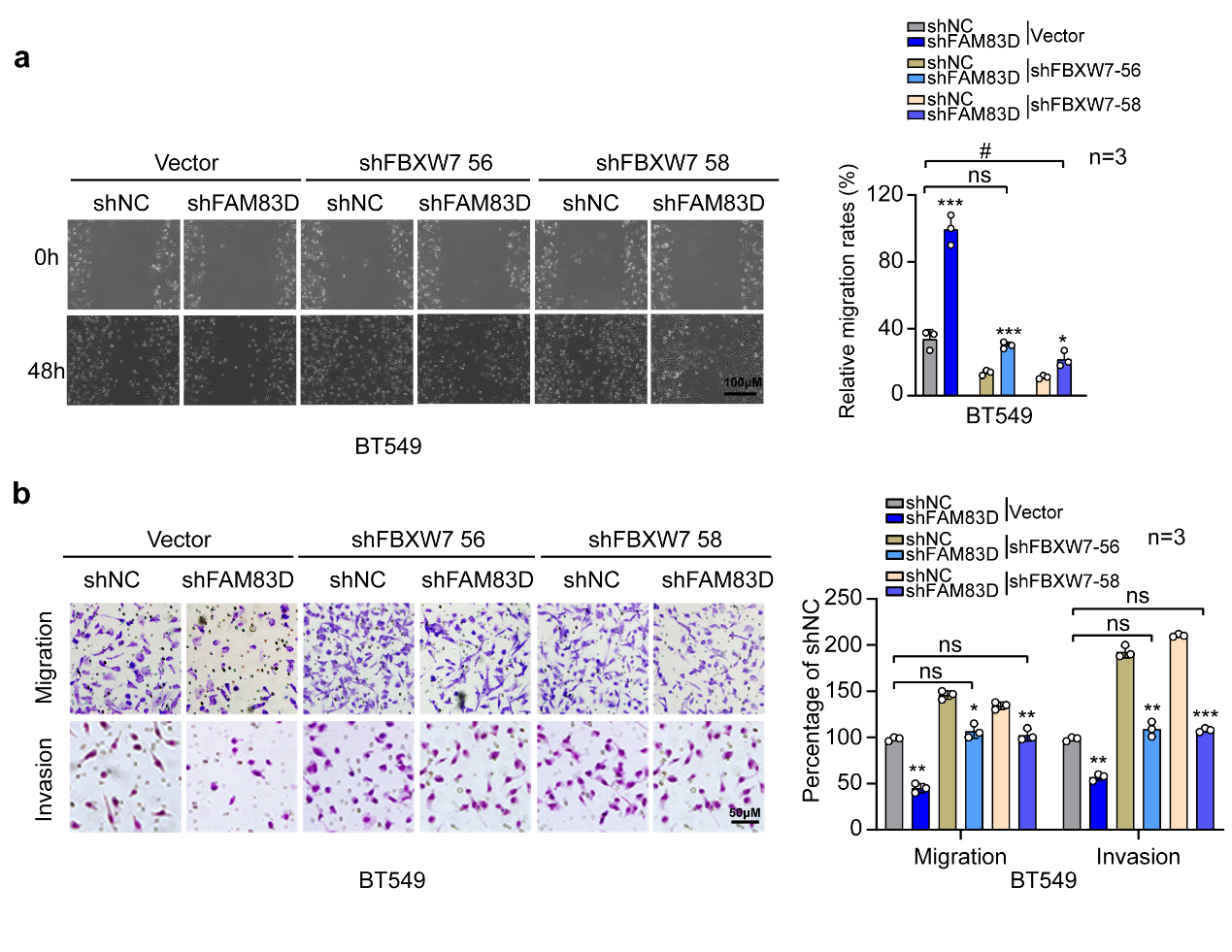
**

**Extended Data Fig. 2 FBXW7 knockdown impairs the inhibitory effects of migration and invasion of BT549 cells induced by FAM83D deficiency. a,** The effects of FBXW7 knockdown on FAM83D deficiency-triggered cell migration inhibition of BT549 cells were examined by wound healing assay. Quantitative analyses were shown in the graphs (n=3). **b,** The effects of FBXW7 knockdown on FAM83D deficiency-triggered cell migration and invasion inhibition of BT549 cells were examined by Matrigel coated or uncoated Transwell assay. Quantitative analyses were shown in the graphs (n=3). Data are presented by mean ± SD. ns: not significant, ^✱^P<0.05, **^✱✱^**P<0.01, **^✱✱✱^**P<0.001 based on the Student’s test.

**
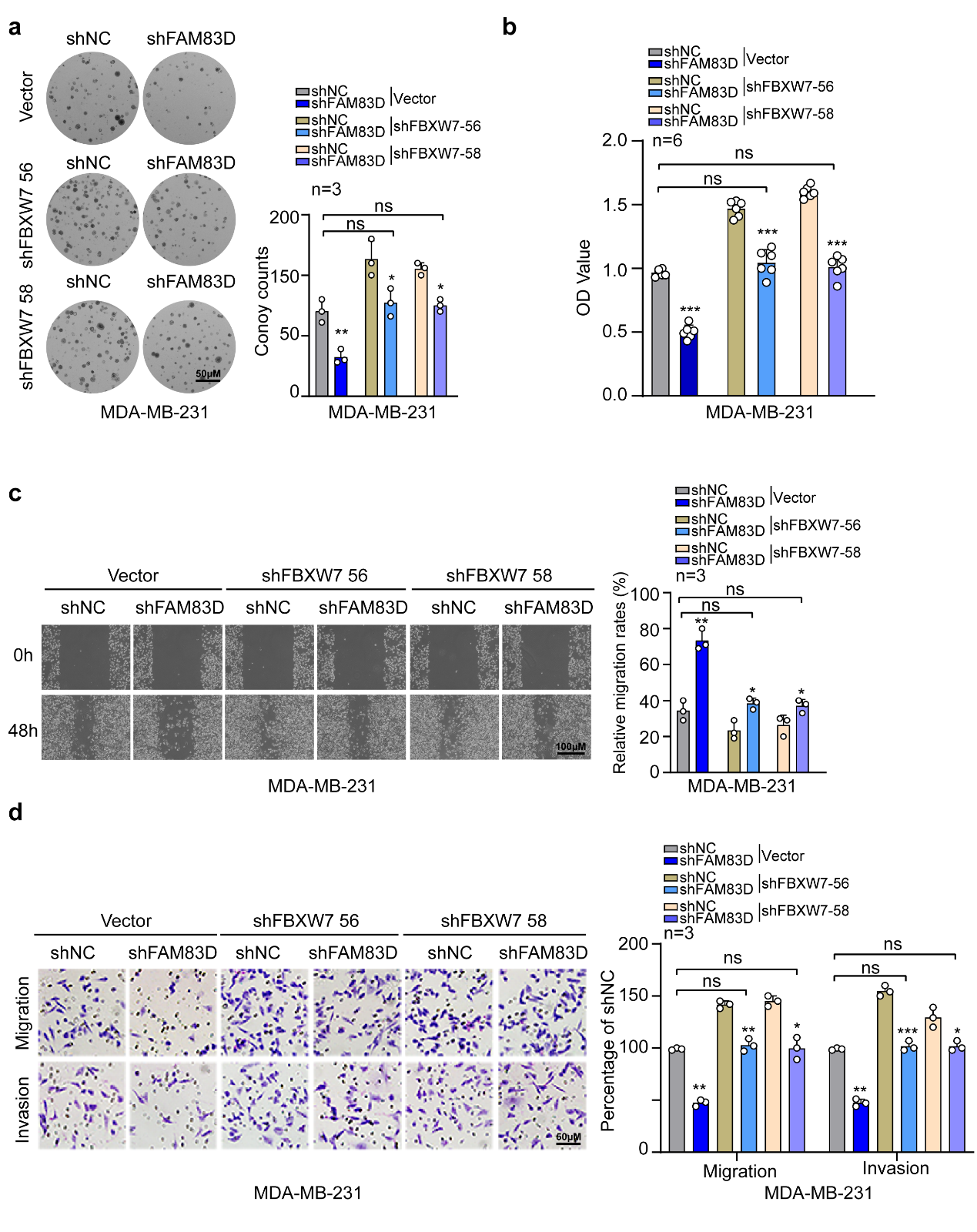
**

**Extended Data Fig. 3 FBXW7 knockdown impairs the inhibitory effects of FAM83D deficiency on cell proliferation, migration and invasion of MDA-MB-231 cells. a,** The effects of FBXW7 knockdown on the inhibitory effects of FAM83D deficiency on MDA-MB-231 cell viability were determined by clonogenic assay. Quantitative analyses were shown in the graphs. **b,** The effects of FBXW7 knockdown on the inhibitory effects of FAM83D deficiency on MDA-MB-231 cell viability were determined by CCK8 assay. **c-d,** The effects of FBXW7 knockdown on the inhibitory effects of FAM83D deficiency on MDA-MB-231 cell migration and invasion were determined by wound-healing assay (c) and Matrigel coated or uncoated Transwell assay (d). Quantitative analyses were shown in the graphs. Data were presented as mean ± SD. ns: not significant. **: p<0.01, ***: p<0.001 based on the Student’s *t*-test.

**
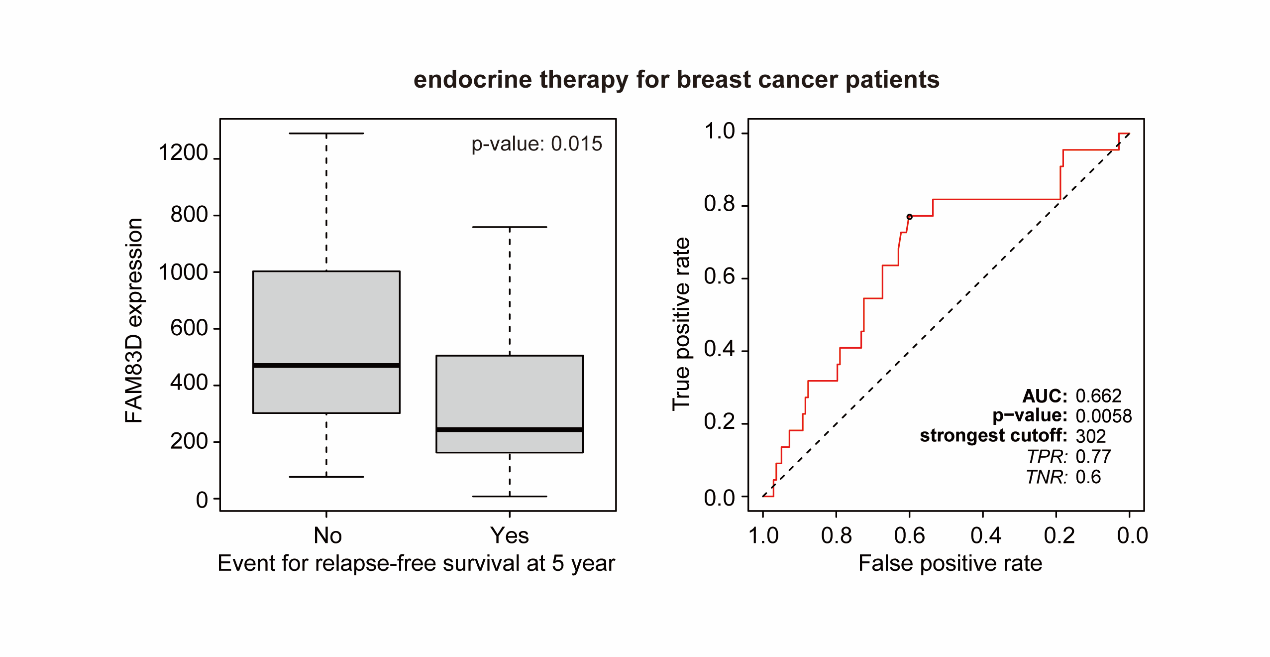
**

**Extended Data Fig. 4 High expression of FAM83D confers resistance to endocrine therapy for breast cancer patients.** FAM83D expression significantly correlated with relapse-free survival (RFS) to endocrine therapy in breast cancer patients. Significant difference in FAM83D expression between two response groups (left panel) and the predictive value of FAM83D expression for RFS to endocrine therapy (right panel) were obtained ROC plotter (<https://www.rocplot.org/>).

**Extended Data Table 1 Primers used in this study**

| Name |  | Sequence（5’-3’） |
| --- | --- | --- |
| FLAG-FAM83D-1-490 | F | CGGAATTCAATGGCGCGGGCCTGTTTG |
|  | R | CGGGATCCTTATTCTGTAGATTGAGTTCCTCGAG |
| FLAG-FAM83D-1-365 | F | CGGAATTCAATGGCGCGGGCCTGTTTG |
|  | R | CGGGATCCTTAAGCCAGCCGCATCCGCA |
| FLAG-FAM83D-1-341 | F | CGGAATTCAATGGCGCGGGCCTGTTTG |
|  | R | CGGGATCCTTAAAACTTGTTGCTGCTCTGGAAG |
| FLAG-FAM83D-1-335 | F | CGGAATTCAATGGCGCGGGCCTGTTTG |
|  | R | CGGGATCCTTAGAAGTGAGACAGGAGTTTGGG |
| FLAG-FAM83D-1-330 | F | CGGAATTCAATGGCGCGGGCCTGTTTG |
|  | R | CGGGATCCTTATTTGGGGCTGATGGGCTTGGA |
| FLAG-FAM83D-350-615 | F | CGGAATTCGCAGTCCAAGGAGCTCACCC |
|  | R | CGGGATCCTTACTGATAGGAAGGATAAAGTGCT |
| FLAG-FAM83D-373-615 | F | CGGAATTCGAAGGCGGACCTGGACCC |
|  | R | CGGGATCCTTACTGATAGGAAGGATAAAGTGCT |
| FLAG-FAM83D  M1 (K340R/F341Y) | F | CAGCAACAGATATGATCACCTCACCAAC |
|  | R | ATCATATCTGTTGCTGCTCTGGAAGTG |
| FLAG-FAM83D  M2 (H343R/L344A) | F | GATCGCGCCACCAACCGAAAACCACAGTC |
|  | R | CGGTTGGTGGCGCGATCAAACTTGTTGC |
| FLAG-FAM83D  M3 (P349A) | F | AAAGCACAGTCCAAGGAGCTCAC |
|  | R | CCTTGGACTGTGCTTTTCGGTTGG |

**Extended Data Table 2 The types, dilutions and sources of antibodies used for western bloting and immunohistochemistry (IHC) analysis**

| **Antibody** |  | **Working dilution** |  | **Working dilution** |  | **Species** | **Source -Cat. Number** |
| --- | --- | --- | --- | --- | --- | --- | --- |
|  |  | **Western blotting** |  | **IHC** |  |  |  |
| FAM83D |  | 1:1000 |  | — |  | Rabbit polyclonal | Abcam  (Cat. No. ab236882) |
| FBXW7 |  | 1:1000 |  | — |  | Rabbit polyclonal | Abcam  (Cat. No. ab192328 ) |
| FBXW7 |  | — |  | 1:50 |  | Rabbit polyclonal | Abcam  (Cat. No. ab84783) |
| CyclinE |  | 1:1000 |  | — |  | Rabbit monoclonal | Abcam  (Cat. No. ab33911) |
| c-Myc |  | 1:2000 |  | — |  | Rabbit monoclonal | Abcam  (Cat. No. ab185656) |
| AuroraA |  | 1:1000 |  | — |  | Mouse monoclonal | Abcam  (Cat. No. ab13824) |
| Myc-tag |  | 1:2000 |  | — |  | Mouse monoclonal | Cell Signaling Technology  (Cat. No. 2276) |
| Flag-tag |  | 1:2000 |  | — |  | Mouse  monoclonal | Sigma  (Cat. No.F1804) |
| HA-tag |  | 1:2000 |  | — |  | Mouse  monoclonal | Sigma  (Cat. No. H3663) |
| β-Actin |  | 1:3000 |  | — |  | fpolyclonal | Cell Signaling Technology  (Cat. No. ab64659) |
