# Supplementary figures and images for: The FBXW7-binding sites on FAM83D are potential targets for cancer therapy
Source: Breast Cancer Res. 2024 Mar 7;26:37. doi: 10.1186/s13058-024-01795-9 (PMC10918900; doi:10.1186/s13058-024-01795-9)

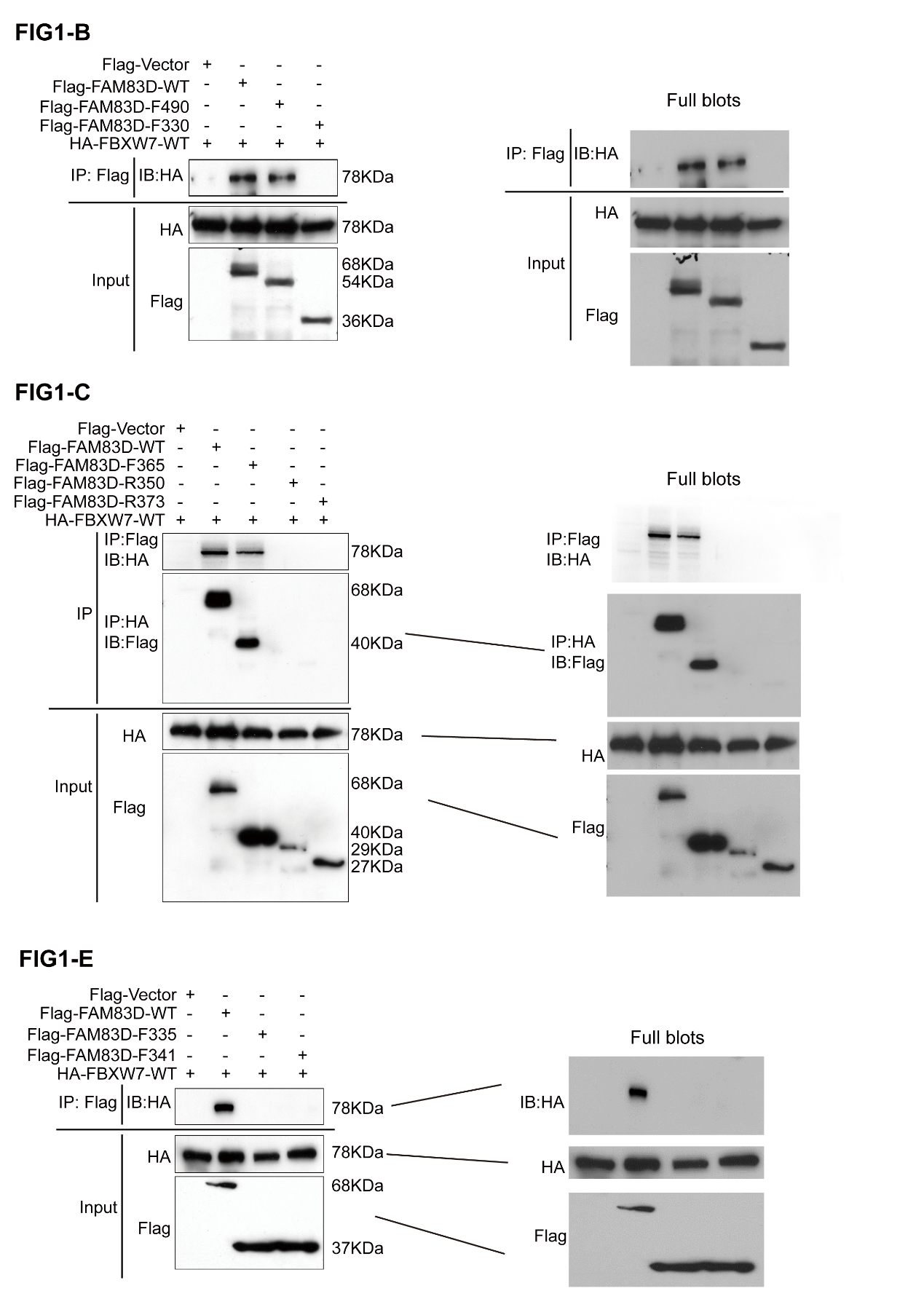


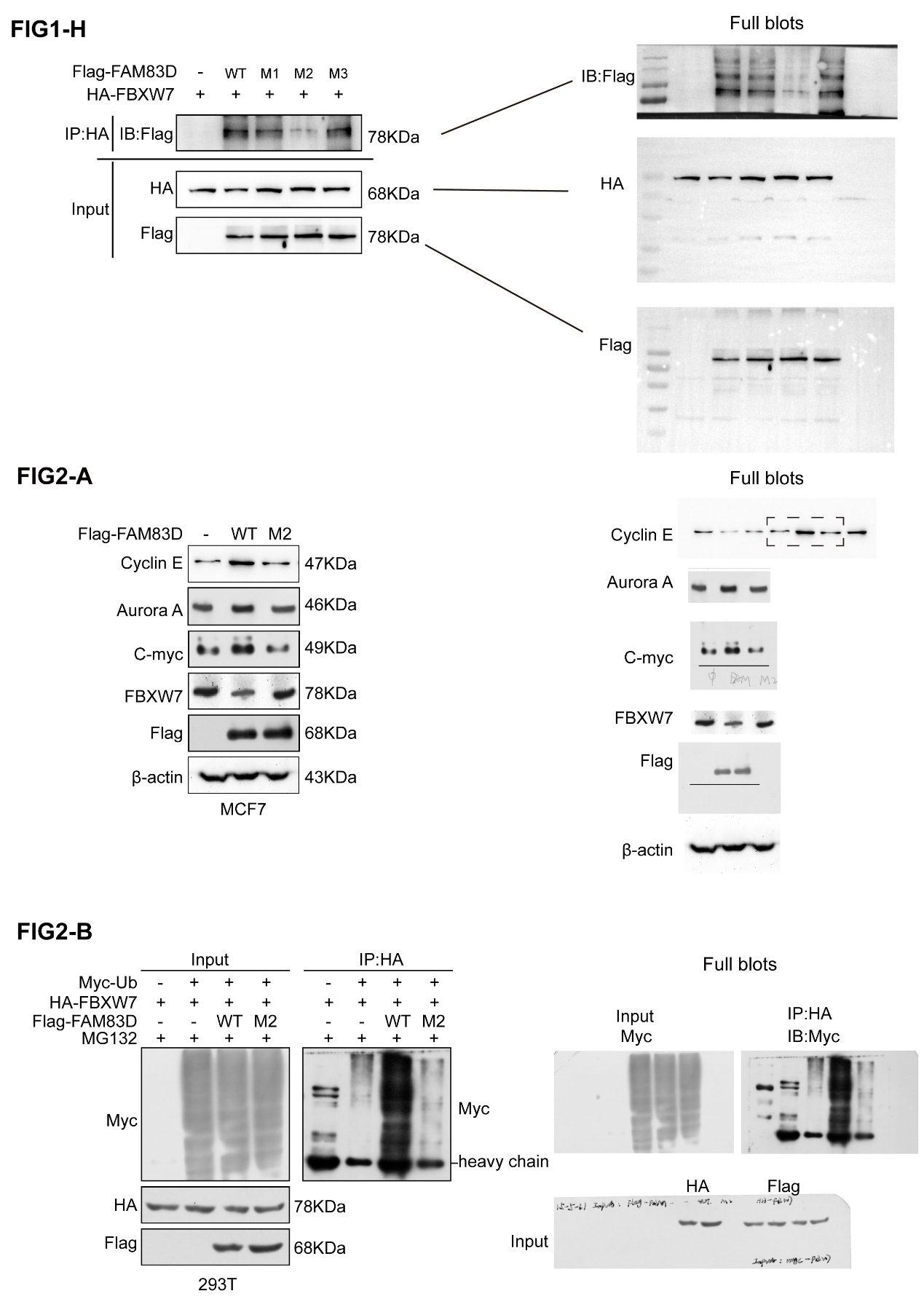


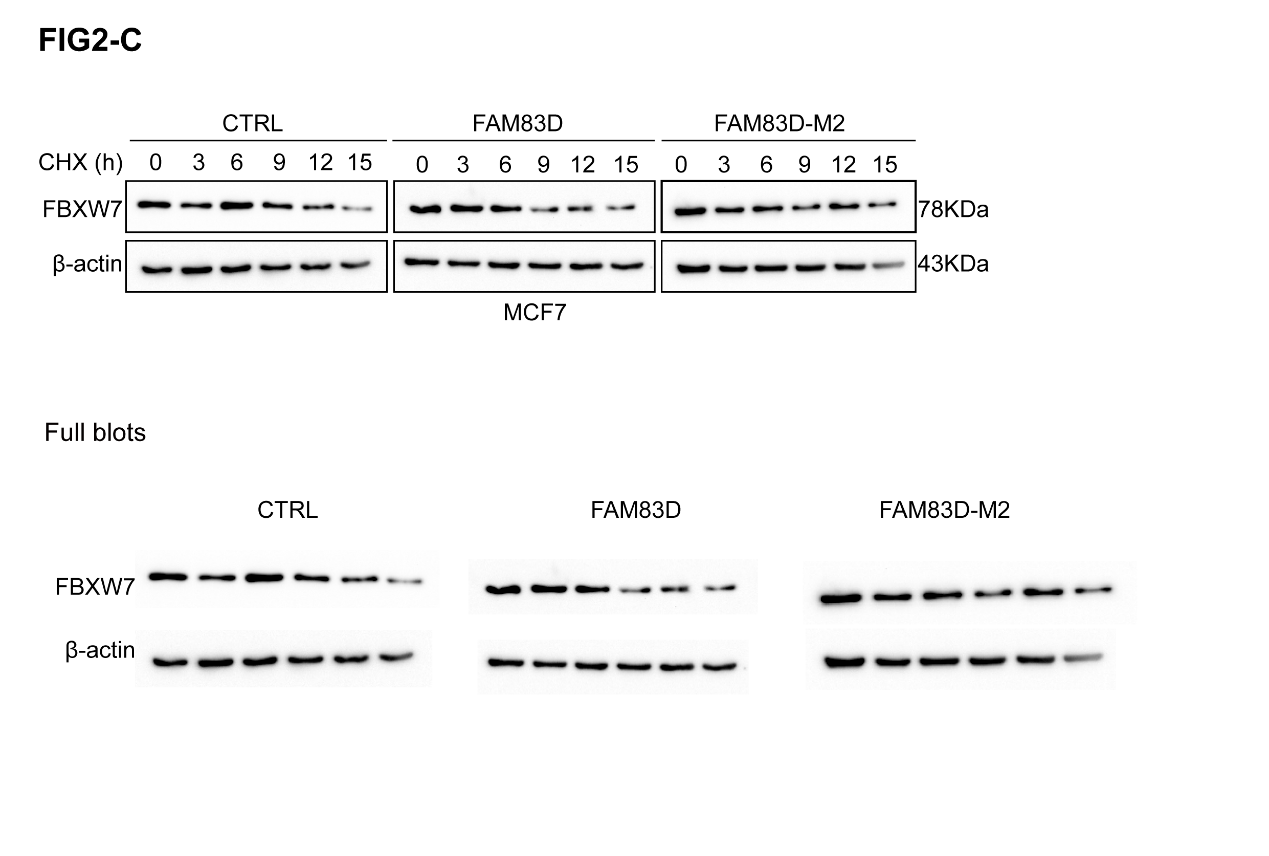


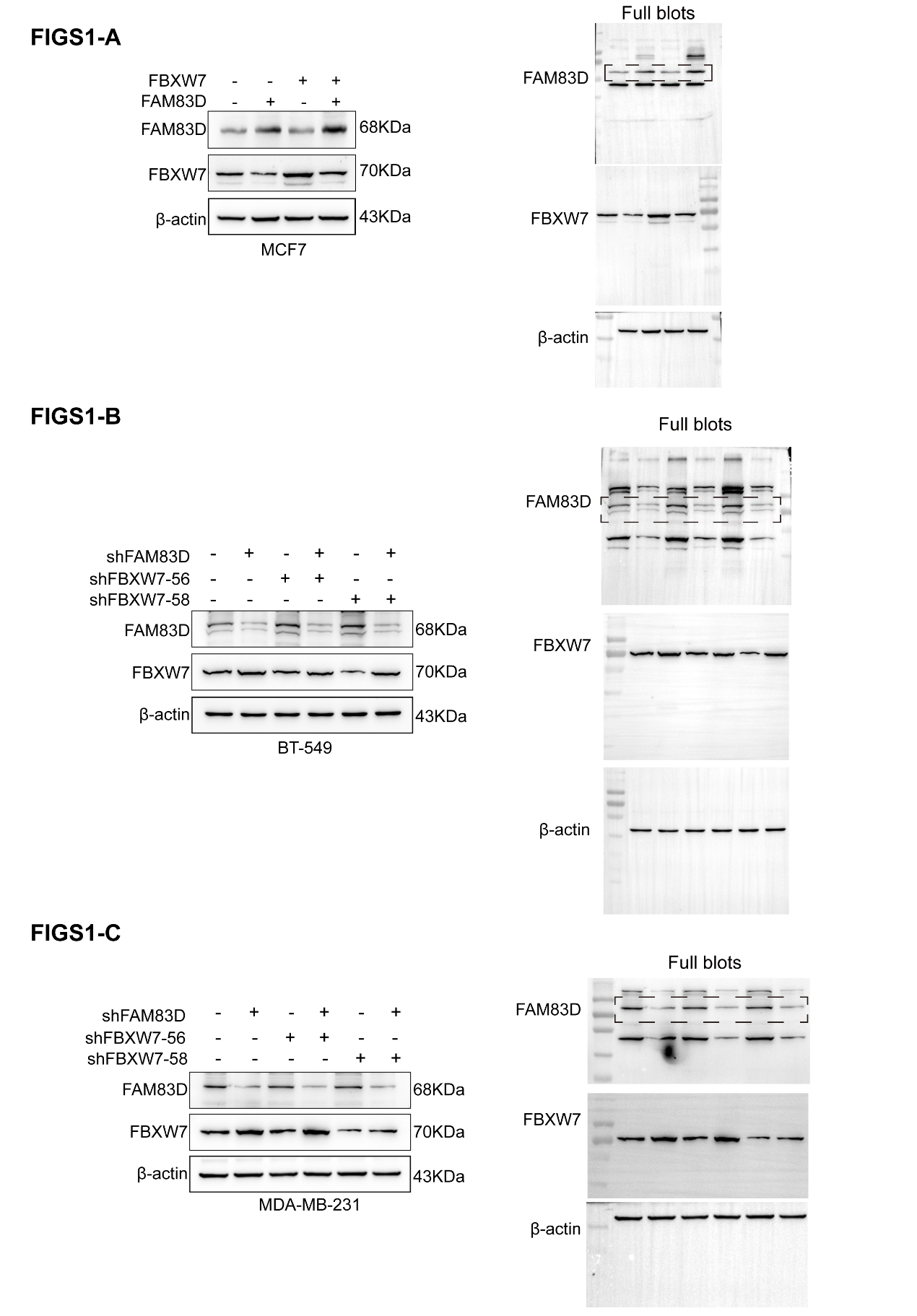

Supplement: Supplementary file 2 — Supplementary Material 2: Unprocessed western blots for different figures [file 13058_2024_1795_MOESM2_ESM.docx]
